# Stimulation of Intestinal Peristalsis by Physiological Means

**Published:** 1911-03

**Authors:** 


### Stimulation of Intestinal Peristalsis by Physiological Means

As has been demonstrated by Zuelzer, Dohrn and Marxer, normal intestinal peristalsis is stimulated by a specific cellular product (hormone) which occurs in substantial quantities chiefly in the spleen. This product is now isolated by special process by the Schering Chemical Works of Berlin, Germany, and marketed under the name of hormonal. It is carefully sterilised and the peristaltic efficacy of each lot is tested by animal experiment.

Hormonal is indicated above all in the various forms of chronic constipation which fail to yield to or are not permanently relieved by the customary treatment. On account of its specific action hormonal can of course in no wise be compared with the cathartics. It is distinguished from the latter by the fact that it causes intestinal peristalsis in a physiological sense and that its action in those cases, which respond to the treatment, is permanent. Even cases of constipation of many years' standing, which have resisted all other forms of treatment, will yield in most instances to a single injection of hormonal, normal intestinal peristalsis being reëstablished followed by spontaneous evacuations, the latter nearly always remaining so for several months. (Dr. Saar of the Second Medical University Clinic, Charite, Berlin, Germany, in *Medizinische Klinik,* 1910, No. 11). The effect is generally noted on the second or third day, in some cases later.

Hormonal which is likewise indicated in ileo-postoperative and peritonitis intestinal paresis, where it often proves the only means of saving the patient's life, is furnished in two forms: (1) hormonal intramuscular, containing ¼ per cent. of beta-eucaine hydrochloride and employed in the treatment of chronic constipation; (2) hormonal intravenous, without beta-eucaine, for the treatment of intestinal paresis and the like.

As already stated, not all cases react favorably to hormonal injections and it is so far not possible to determine definitely which class of cases responds electively. Favorable results with hormonal have also been obtained in a great number of cases of intestinal atony and their prophylactic employment in abdominal operations appears entirely logical.

